# Risk factors for an acute exacerbation of idiopathic pulmonary fibrosis

**DOI:** 10.1186/s12931-016-0400-1

**Published:** 2016-07-11

**Authors:** Tomoyuki Kakugawa, Noriho Sakamoto, Shuntaro Sato, Hirokazu Yura, Tatsuhiko Harada, Shota Nakashima, Atsuko Hara, Keishi Oda, Hiroshi Ishimoto, Kazuhiro Yatera, Yuji Ishimatsu, Yasushi Obase, Shigeru Kohno, Hiroshi Mukae

**Affiliations:** Department of Respiratory Medicine, Unit of Translational Medicine, Nagasaki University Graduate School of Biomedical Sciences, Nagasaki, Japan; Clinical Research Center, Nagasaki University Hospital, Nagasaki, Japan; Division of Biostatistics, Kurume University School of Medicine, Fukuoka, Japan; Department of Respiratory Medicine, School of Medicine, University of Occupational and Environmental Health, Kitakyushu, Japan; Department of Cardiopulmonary Rehabilitation Sciences, Nagasaki University Graduate School of Biomedical Sciences, Nagasaki, Japan

**Keywords:** Bronchoalveolar lavage, Krebs von den Lungen-6, Surfactant protein-D, Lactate dehydrogenase, GAP (gender, age, Physiology) stage, Cardiovascular disease

## Abstract

**Background:**

Acute exacerbations of idiopathic pulmonary fibrosis are major causes of morbidity and mortality among patients with idiopathic pulmonary fibrosis. However, acute exacerbations remain unpredictable. The aim of this study was to investigate risk factors for acute exacerbations of idiopathic pulmonary fibrosis.

**Methods:**

We performed a retrospective cohort study of patients with idiopathic pulmonary fibrosis who visited our institutions from January 1999 to September 2014. We investigated risk factors for acute exacerbations in patients with idiopathic pulmonary fibrosis diagnosed retrospectively based on the official 2011 idiopathic pulmonary fibrosis ATS/ERS/JRS/ALAT Update Statement.

**Results:**

The idiopathic pulmonary fibrosis study cohort included 65 subjects. The median follow-up period was 2.6 years. During follow-up, 24 patients (36.9 %) experienced acute exacerbations. A Kaplan-Meier curve demonstrated that the 1-year, 2-year, and 3-year incidences of acute exacerbation were 9.6, 19.2 and 31.0 %, respectively. Acute exacerbation exerted a significant impact on overall survival among those with the disease. A log-rank test showed that baseline cardiovascular diseases, higher GAP (gender, age, physiology) stage (≥II), higher serum lactate dehydrogenase level (≥180 U/L), higher serum surfactant protein-D level (≥194.7 ng/mL), higher neutrophil (≥1.77 %) and eosinophil (≥3.21 %) percentages in bronchoalveolar lavage fluid samples, and treatment with an immunosuppressive agent after diagnosis were associated with poor acute exacerbation-free probability. In the Cox analysis adjusted for treatment with an immunosuppressive agent, baseline cardiovascular diseases, higher GAP stage (≥II), and higher eosinophil percentage (≥3.21 %) in bronchoalveolar lavage fluid samples were predictors of an acute exacerbation of idiopathic pulmonary fibrosis.

**Conclusions:**

This study demonstrated that baseline cardiovascular diseases, higher GAP stage (≥II), and higher eosinophil percentage (≥3.21 %) in bronchoalveolar lavage fluid samples were associated with the onset of an acute exacerbation of idiopathic pulmonary fibrosis.

## Background

Idiopathic pulmonary fibrosis (IPF) is the most common form of idiopathic interstitial pneumonia [1, 2], and the median survival from the time of diagnosis is approximately 3 years [2]. The clinical course of individual patients with IPF is highly variable and unpredictable. IPF is not a uniform, clinically dynamic disease; there is a wide spectrum of disease courses, including stability or slow progression over a period of years, rapid deterioration, and even periods of relative stability punctuated by events causing rapid decline [3–5]. This acute worsening of the disease is sometimes attributed to identifiable conditions such as pneumonia and heart failure. However, many of these events occur without an identifiable cause, and are called acute exacerbations (AEs) of IPF [4]. AE-IPF is a major cause of morbidity and mortality in patients with IPF [3, 6–8]. In 553 Japanese patients with IPF, the most common cause of death was AE with a frequency of 40 % [8], which was similar to the 46 % value found in a previous study by Jeon and coworkers in Korea [6]. There is no proven treatment for AE-IPF. Patients with AEs have poor outcomes, with mortality exceeding 60 % during hospital admission; among those who survive, there is >90 % mortality within 6 months after discharge [3, 4]. Therefore, elucidating the pathogenesis of AE-IPF and predicting AE are important and challenging issues.

Kondoh et al. reported that a higher body mass index, higher modified Medical Research Council scale score, and decline in forced vital capacity (FVC) at 6 months after the diagnosis of IPF were independent risk factors for AE-IPF [7]. In one study, a comparison of clinical features between IPF patients with and without an AE-IPF showed no difference in age, smoking status, oxygenation, or lung function at the time of diagnosis [9]. Risk factors for AE-IPF reported by Song et al. [3] include a lower FVC at baseline as well as never having smoked. Pulmonary hypertension at baseline was also reported to be associated with a significant risk of a subsequent AE-IPF [10]. More recently, Kondoh et al. reported that a rapid decline in vital capacity percentage (≥10 % within 6 months after the diagnosis of IPF) and high baseline alveolar-arterial difference in oxygen (A-a DO_2_) value might be risk factors for an AE-IPF over the 52 weeks of the study [11]. However, studies regarding risk factors for AE-IPF are still limited, and AEs remain unpredictable. In addition, serum biomarkers and cellular analyses of bronchoalveolar lavage (BAL) fluid are still of unclear predictive value for AE-IPF. Furthermore, in most previous studies investigating risk factors for AE-IPF, the diagnosis of IPF was made based on diagnostic criteria developed before the official IPF ATS/ERS/JRS/ALAT Update Statement in 2011 [2].

The aim of this study was to investigate risk factors for AE-IPF, focusing on the significance of serum markers and cellular analyses of BAL fluid in patients with IPF who were diagnosed based on the official 2011 IPF ATS/ERS/JRS/ALAT Update Statement [2].

## Methods

### Study population

We performed a retrospective cohort study of patients with IPF who visited Nagasaki University Hospital, Nagasaki, Japan, or University Hospital of Occupational and Environmental Health, Kitakyushu, Japan, from January 1999 to September 2014. Diagnoses were made retrospectively according to the official ATS/ERS/JRS/ALAT statement [2]. Patients with underlying connective tissue disease, occupational or environmental exposure, or a histopathologic pattern on surgical lung biopsy other than usual interstitial pneumonia (UIP) were excluded from the study. At our institution, patients with suspected IPF routinely underwent bronchoscopy with BAL at the time of initial diagnosis, unless the patient’s status contraindicated to the need for bronchoscopy. Patients whose initial manifestation was an AE, patients without initial BAL fluid evaluations, and patients lost to follow-up within 90 days were excluded. Twelve of 65 (18.5 %) IPF diagnoses were confirmed pathologically in multiple lobes by open lung biopsy or video-assisted thoracoscopic surgery. AE-IPF was defined using the following previously reported criteria with a slight modification: (1) a previous diagnosis of IPF; (2) unexplained worsening of dyspnea within the past 30 days; (3) high-resolution computed tomography (HRCT) with new findings of bilateral ground-glass opacity or consolidation; and (4) the absence of an alternative explanation such as infection, pulmonary embolism, pneumothorax, or heart failure [2, 4]. The study protocol was approved by the Institutional Review Boards of Nagasaki University Hospital and University Hospital of Occupational and Environmental Health. The patients’ approval or informed consent was not required for a retrospective review of their records, pursuant to the ethical guidelines of the Japanese Ministry of Health, Labor, and Welfare.

### Covariates

Data regarding patient characteristics were collected from clinical notes recorded at the time of the initial diagnosis of IPF and included age, sex, smoking history, partial pressure of oxygen in arterial blood (PaO_2_)/fraction of inspired oxygen (FiO_2_) ratio (P/F ratio), A-a DO_2_, hematologic data, pulmonary function tests, and BAL fluid cellular constituents. Data regarding serum concentrations of C-reactive protein (CRP), lactate dehydrogenase (LDH), Krebs von den Lungen-6 (KL-6), and surfactant protein (SP)-D were also collected from clinical notes recorded at the time of diagnosis. Gender-Age-Physiology (GAP) stage and composite physiologic index (CPI) were calculated as described previously [12, 13].

### BAL and cell preparation

Bronchoscopy and BAL were performed as described previously [14].

### Clinical and outcome assessments

In the AE analysis, subjects were censored if they (1) had not experienced an AE by September 30, 2014, or (2) were lost to follow-up. In the survival analysis, subjects were censored if they (1) were still alive on September 30, 2014, or (2) lost to follow-up. AE-free probability and survival probability were calculated from the time of the initial IPF diagnosis to censoring.

### Statistical analysis

Baseline characteristics including demographics, baseline pulmonary function, respiratory parameters, hematologic data, serum markers, and BAL fluid findings were compared between patients who experienced an AE during the study period and those who did not. Data are described as frequencies for categorical variables and as the median and interquartile range (IQR) for quantitative variables. Associations between variables were assessed with Fisher’s exact test for categorical variables and Wilcoxon’s rank-sum test for quantitative variables. The cumulative AE incidence was estimated with the Kaplan-Meier method. The association between survival probability and AE was evaluated by the log-rank test. A log-rank test was used to evaluate associations among smoking status, cardiovascular diseases, GAP stage, biomarkers (LDH, KL-6, and SP-D), BAL fluid cellular profiles (percentages of lymphocytes, neutrophils, and eosinophils), treatment after diagnosis, and AE-free probability because the test is a simple method that did not require satisfying some assumptions. Cox’s proportional hazards regression models were used to evaluate hazard ratios (HRs) and adjust confounders. It was considered appropriate to determine a cut-off level instead of using continuous data because some factors did not satisfy the assumption that the log hazard increased linearly with the covariate. The optimum cut-off values for predicting AE incidence at the 3-year follow-up were determined by maximizing the Youden index [15] calculated from a time-dependent receiver operating characteristic (ROC) analysis with the R package timeROC [16]. We reported the results at the 3-year follow-up, as this was the interval with the most dramatic relationship between biomarkers, BAL fluid cellular profiles, and AE incidence. We evaluated HRs, 95 % confidence intervals (95 % CIs), and *P* values for each factor with an unadjusted Cox analysis. Because of the small number of AEs and deaths included in this study, it was considered inappropriate to adjust more than two factors in a multiple Cox analysis to evaluate the relative hazards of AE and death. Accordingly, we adjusted for treatment with an immunosuppressive agent in the multiple Cox analysis, which was found to be a significant risk factor for an AE-IPF in the log-rank test and unadjusted Cox analysis in this study. All tests were two-sided, and a *P* value less than 0.05 was considered statistically significant. All statistical analyses were performed using SAS version 9.4 (SAS Institute Inc., Cary, NC, USA) and R version 3.1.0.

## Results

### Subject characteristics

The IPF study cohort included 65 subjects. There were 51 men and 14 women with a median age of 69 years (IQR, 62 to 73 years). There were 13 never smokers, 42 former smokers, and 10 current smokers. The median follow-up period was 2.6 years (IQR, 1.3 to 4.2 years). The median survival time (MST) for all subjects was 4.5 years from the time of the initial visit.

### AE incidence

**Fig. 1:**
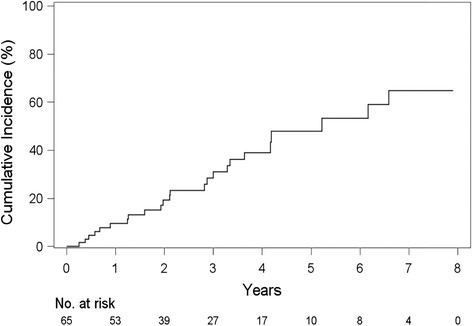
Incidence of acute exacerbation of idiopathic pulmonary fibrosis. The Kaplan-Meier curve demonstrates that the 1-year, 2-year, and 3-year incidences of AE-IPF were 9.6, 19.2 and 31.0 %, respectively

During the follow-up period, 24 patients (36.9 %) experienced AEs. Eighteen patients (27.7 %) were lost to follow-up. The Kaplan-Meier curve demonstrated that the 1-year, 2-year, and 3-year incidences of AE-IPF were 9.6, 19.2 and 31.0 %, respectively (Fig. 1).

### Demographic, physiological, gas exchange, and laboratory data and BAL fluid findings between patients with an AE (AE group) and those without an AE (Non-AE group)

No significant differences were observed in sex or age distributions between the groups. Patients who experienced an AE had a lower total lung capacity than those in the Non-AE group. Gas exchange and laboratory data and BAL fluid findings were similar between the groups (Table 1).

**Table 1:** Comparisons of baseline characteristics between patients with and without an acute exacerbation

	Non-AE		AE		*P* Value ^a^
	(*N* = 41)		(*N* = 24)		
Age, median (IQR), y	69.0	(62.0 to 73.0)	68.0	(62.0 to 73.5)	0.995
Sex, No. (%)					
Men	31	(75.6)	20	(83.3)	1.000
Women	10	(24.4)	4	(16.7)	
Smoking, No. (%)					
S	7	(17.1)	3	(12.5)	1.000
Ex	25	(61.0)	17	(70.8)	
N	9	(22.0)	4	(16.7)	
Smoking index, pack-years	35.8	(1.0 to 52.3)	31.3	(15.0 to 50.0)	0.940
Cardiovascular diseases, No. (%)					
Yes	10	(24.4)	8	(33.3)	1.000
No	31	(75.6)	16	(66.7)	
Atopic diseases, No. (%)					
Yes	3	(7.3)	1	(4.2)	1.000
No	38	(92.7)	23	(95.8)	
Baseline pulmonary function					
VC, median (IQR), % predicted	81.0	(64.1 to 95.0)	69.2	(59.5 to 83.0)	0.074
FVC, median (IQR), % predicted	80.4	(59.0 to 93.9)	70.5	(59.9 to 82.1)	0.055
TLC, median (IQR), % predicted	75.9	(63.0 to 90.0)	66.1	(54.0 to 77.0)	0.020
DLCO, median (IQR), % predicted	58.0	(46.1 to 72.5)	46.2	(41.0 to 72.3)	0.276
KCO, median (IQR), % predicted	69.5	(60.8 to 85.0)	73.0	(57.7 to 78.0)	0.866
GAP stage, No. (%)					
I	25	(69.4)	10	(45.5)	1.000
II	9	(25.0)	12	(54.5)	
III	2	(5.6)	0	(0.0)	
CPI	37.3	(22.2 to 47.6)	47.7	(30.5 to 54.9)	0.190
Respiratory parameters ^b^					
PaO_2_, median (IQR), mm Hg	80.7	(75.3 to 91.0)	81.0	(75.2 to 94.5)	0.810
A-a DO_2_, median (IQR), mm Hg	17.5	(3.8 to 26.2)	17.2	(4.1 to 25.2)	0.889
Hematologic data					
WBC, median (IQR),/mm^3^	6700	(5800 to 8100)	7350	(5750 to 9300)	0.442
Neutrophils, median (IQR), %	62.8	(57.0 to 69.4)	63.9	(58.0 to 69.0)	0.897
Eosinophils, median (IQR), %	3.0	(2.0 to 4.7)	2.9	(1.8 to 4.4)	0.545
Serum markers					
CRP, median (IQR), mg/dL	0.31	(0.06 to 0.69)	0.29	(0.13 to 0.64)	0.688
LDH, median (IQR), U/L	235	(187.0 to 255.0)	247	(223.5 to 296.0)	0.082
KL-6, median (IQR), U/mL	912	(595.0 to 1802.0)	1258	(744.5 to 2006.0)	0.246
SP-D, median (IQR), ng/mL	203	(131.0 to 342.0)	269	(178.6 to 343.0)	0.285
BAL fluid findings					
Total cell count, median (IQR), ×10^5^/mL	2.50	(1.40 to 3.67)	3.21	(2.22 to 3.97)	0.099
Macrophages, median (IQR), %	80.2	(62.5 to 88.9)	76.6	(67.3 to 84.8)	0.492
Lymphocytes, median (IQR), %	6.8	(3.9 to 11.4)	11.6	(3.2 to 17.1)	0.649
Neutrophils, median (IQR), %	6.7	(2.9 to 13.3)	7.0	(3.2 to 10.5)	0.807
Eosinophils, median (IQR), %	3.3	(1.2 to 5.4)	4.6	(2.1 to 7.0)	0.259
CD4/8 ratio, median (IQR)	1.90	(0.86 to 3.70)	1.10	(0.60 to 1.90)	0.065
Treatment after the diagnosis					
Steroid, No. (%)					
yes	7	(17.1)	8	(33.3)	1.000
No	34	(82.9)	16	(66.7)	
Immunosuppressive agent ^c^, No. (%)					
Yes	3	(7.3)	6	(25.0)	1.000
No	38	(92.7)	18	(75.0)	
Steroid with immunosuppressive agent, No. (%)					
Yes	3	(7.3)	5	(20.8)	1.000
No	38	(92.7)	19	(79.2)	
Pirfenidone, No. (%)					
Yes	8	(19.5)	6	(25.0)	1.000
No	33	(80.5)	18	(75.0)	
NAC, No. (%)					
Yes	8	(19.5)	5	(20.8)	1.000
No	33	(80.5)	19	(79.2)	

*Abbreviations*: *AE* acute exacerbation, *N* number of patients, *IQR* interquartile range, *S/Ex/N* current smoker/ex-smoker/nonsmoker, *VC* vital capacity, *FVC* forced vital capacity, *TLC* total lung capacity, *DLCO* diffusing capacity for carbon monoxide, *KCO* carbon monoxide transfer coefficient, *GAP* gender-age-physiology, *CPI* composite physiologic index, *P/F ratio* PaO_2_/fraction of inspired oxygen ratio, *A-a DO*_*2*_ alveolar-arterial difference in oxygen, *WBC* white blood cell count, *CRP* C-reactive protein, *LDH* lactate dehydrogenase, *KL-6* Krebs von den Lungen-6, *SP-D* surfactant protein-D, *BAL* bronchoalveolar lavage, *CD4/8 ratio* CD4/CD8 lymphocyte subset ratio, *NAC* inhaled N-acetylcysteine

Data are presented as the median (interquartile range)

^a^Wilcoxon’s rank-sum test was performed for continuous variables, and Fisher’s exact test was performed for categorical variables

^b^One patient in the Non-AE group required oxygen (1 L/min O_2_ via a nasal cannula) at the time of sample collection. All other results were obtained at room air

^c^Immunosuppressive agents included cyclosporine (*N* = 7) and cyclophosphamide (*N* = 2)

### Impact of AE on overall survival

**Fig. 2:**
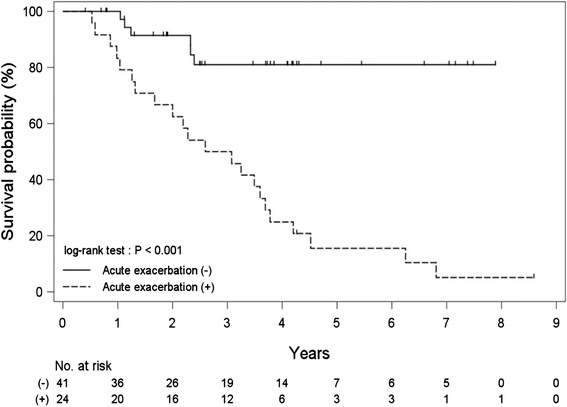
Impact of acute exacerbation on overall survival. There was a significant difference in survival between patients who experienced an acute exacerbation (median survival time, 2.8 years) and those who did not (median survival time, not calculable) (log-rank test, *P* < 0.001)

AE exerted a significant impact on overall survival in IPF. There was a significant difference in survival between patients who experienced an AE (MST, 2.8 years) and those who did not (MST, not calculable) (log-rank test, *P* < 0.001) (Fig. 2).

### Risk factors for AE

According to the ROC curve analysis, the optimum cut-off levels to predict an AE-IPF were 180 U/L for LDH, 946 U/mL for KL-6, 194.7 ng/mL for SP-D; and 10.15 1.77 and 3.21 % for lymphocyte, neutrophil, and eosinophil percentages in BAL fluid samples, respectively. A log-rank test showed that baseline cardiovascular diseases, higher GAP stage (≥II), higher serum LDH level (≥180 U/L), higher serum SP-D level (≥194.7 ng/mL), higher neutrophil (≥1.77 %) and eosinophil (≥3.21 %) percentages in BAL fluid samples, and treatment with an immunosuppressive agent after diagnosis were associated with poor AE-free probability (*P* = 0.042, 0.002, 0.048, 0.014, 0.048, 0.009, and 0.009, respectively) (Table 2). In the unadjusted Cox analysis, treatment with an immunosuppressive agent, baseline cardiovascular diseases, higher GAP stage (≥II), higher serum SP-D level (≥194.7 ng/mL), and a higher eosinophil percentage in BAL fluid samples (≥3.21 %) were predictors of an AE-IPF (HR [95 % CI], 3.35 [1.27–8.84], 2.49 [1.00–6.19], 3.88 [1.54–9.80], 3.17 [1.21–8.31], and 3.27 [1.29–8.29], respectively). In the Cox analysis adjusted for treatment with an immunosuppressive agent, baseline cardiovascular diseases, higher GAP stage (≥II), and higher eosinophil percentage (≥3.21 %) in BAL fluid samples were predictors of an AE-IPF (HR [95 % CI], 3.21 [1.24–8.32], 3.23 [1.22–8.51], and 2.89 [1.11–7.51], respectively) (Table 3).

**Table 2:** Log-rank test for risk factors for an acute exacerbation of idiopathic pulmonary fibrosis

	Non-AE (*N* = 41)	AE (*N* = 24)	log-rank *P* Value
Smoking			
S or Ex	32	20	0.918
N	9	4	
Cardiovascular diseases			
Yes	10	8	0.042
No	31	16	
GAP stage			
II or III	11	12	0.002
I	25	10	
Serum markers			
LDH			
≥ 180	32	22	0.048
< 180	9	2	
KL-6			
≥ 946	18	16	0.292
< 946	21	8	
SP-D			
≥ 194.7	22	18	0.014
< 194.7	17	6	
BAL fluid findings			
Lymphocytes			
≥ 10.15	13	13	0.085
< 10.15	28	11	
Neutrophils			
≥ 1.77	34	22	0.048
< 1.77	7	2	
Eosinophils			
≥ 3.21	21	17	0.009
< 3.21	20	7	
Treatment after the diagnosis			
Steroid			
Yes	7	8	0.443
No	34	16	
Immunosuppressive agent			
Yes	3	6	0.009
No	38	18	
Pirfenidone			
Yes	8	6	0.963
No	33	18	
NAC			
Yes	8	5	0.547
No	33	19	

*Abbreviations*: *AE* acute exacerbation, *N* number of patients, *S/Ex/N* current smoker/ex-smoker/nonsmoker, *GAP* gender-age-physiology, *LDH* lactate dehydrogenase, *KL-6* Krebs von den Lungen-6, *SP-D* surfactant protein-D, *BAL* bronchoalveolar lavage, *NAC* inhaled N-acetylcysteine

**Table 3:** Cox analysis for risk factors for an acute exacerbation of idiopathic pulmonary fibrosis

	HR	(95 % CI)	*P* Value
Treatment after the diagnosis			
Immunosuppressive agent			
Unadjusted	3.35	(1.27 to 8.84)	0.014
Cardiovascular diseases			
Unadjusted	2.49	(1.00 to 6.19)	0.049
Adjusted^a^	3.21	(1.24 to 8.32)	0.016
GAP stage (≥II)			
Unadjusted	3.88	(1.54 to 9.80)	0.004
Adjusted^a^	3.23	(1.22 to 8.51)	0.018
Serum markers			
LDH, U/L, Cut off point = 180			
Unadjusted	4.00	(0.92 to 17.46)	0.065
Adjusted^a^	3.49	(0.78 to 15.57)	0.101
KL-6, U/mL, Cut off point = 946			
Unadjusted	1.57	(0.67 to 3.68)	0.296
Adjusted^a^	1.53	(0.65 to 3.59)	0.326
SP-D, ng/mL, Cut off point = 194.7			
Unadjusted	3.17	(1.21 to 8.31)	0.019
Adjusted^a^	2.69	(0.98 to 7.42)	0.056
BAL fluid findings			
Lymphocytes, %, Cut off point = 10.15			
Unadjusted	2.01	(0.89 to 4.52)	0.091
Adjusted^a^	2.03	(0.90 to 4.57)	0.087
Neutrophils, %, Cut off point = 1.77			
Unadjusted	4.04	(0.92 to 17.79)	0.065
Adjusted^a^	3.54	(0.79 to 15.94)	0.100
Eosinophils, %, Cut off point = 3.21			
Unadjusted	3.27	(1.29 to 8.29)	0.013
Adjusted^a^	2.89	(1.11 to 7.51)	0.029

*Abbreviations*: *HR* hazard ratio, *CI* confidence interval, *GAP* gender-age-physiology, *LDH* lactate dehydrogenase, *KL-6* Krebs von den Lungen-6, *SP-D* surfactant protein-D, *BAL* bronchoalveolar lavage

^a^Adjusted for immunosuppressive agent therapy

### Prognostic factors for overall survival from the initial IPF diagnosis

In the unadjusted Cox analysis, higher baseline GAP stage (≥II), higher serum SP-D level (≥194.7 ng/mL), and higher eosinophil percentage (≥3.21 %) in BAL fluid samples were significant prognostic factors for IPF (HR [95 % CI], 4.02 [1.68–9.64], 3.49 [1.36–8.94], and 2.42 [1.03–5.68], respectively). In the Cox analysis adjusted for treatment with an immunosuppressive agent, baseline cardiovascular diseases, higher GAP stage (≥II), and higher serum SP-D level (≥194.7 ng/mL) were significant prognostic factors for IPF (HR [95 % CI], 2.37 [1.03–5.44], 3.63 [1.47–8.93], and 3.21 [1.21–8.48]) (Table 4).

**Table 4:** Cox analysis for prognostic factors for overall survival from the idiopathic pulmonary fibrosis initial diagnosis

	HR	(95 % CI)	*P* Value
Treatment after diagnosis			
Immunosuppressive agent			
Unadjusted	2.40	(0.96 to 6.02)	0.062
Cardiovascular diseases			
Unadjusted	2.08	(0.91 to 4.54)	0.086
Adjusted^a^	2.37	(1.03 to 5.44)	0.042
GAP stage (≥II)			
Unadjusted	4.02	(1.68 to 9.64)	0.002
Adjusted^a^	3.63	(1.47 to 8.93)	0.005
Serum markers			
LDH, U/L, Cut off point = 180			
Unadjusted	2.49	(0.74 to 8.37)	0.139
Adjusted^a^	2.24	(0.66 to 7.63)	0.198
KL-6, U/mL, Cut off point = 946			
Unadjusted	1.46	(0.67 to 3.18)	0.340
Adjusted^a^	1.45	(0.67 to 3.17)	0.345
SP-D, ng/mL, Cut off point = 194.7			
Unadjusted	3.49	(1.36 to 8.94)	0.009
Adjusted^a^	3.21	(1.21 to 8.48)	0.019
BAL fluid findings			
Lymphocytes, %, Cut off point = 10.15			
Unadjusted	1.96	(0.93 to 4.14)	0.077
Adjusted^a^	1.99	(0.94 to 4.21)	0.072
Neutrophils, %, Cut off point = 1.77			
Unadjusted	2.48	(0.73 to 8.45)	0.147
Adjusted^a^	2.21	(0.63 to 7.69)	0.213
Eosinophils, %, Cut off point = 3.21			
Unadjusted	2.42	(1.03 to 5.68)	0.043
Adjusted^a^	2.20	(0.92 to 5.25)	0.075

*Abbreviations*: *HR* hazard ratio, *CI* confidence interval, *GAP* gender-age-physiology, *LDH* lactate dehydrogenase, *KL-6* Krebs von den Lungen-6, *SP-D* surfactant protein-D, *BAL* bronchoalveolar lavage

^a^Adjusted for immunosuppressive agent therapy

## Discussion

This study examined associations between patient characteristics, serum markers, and BAL fluid cellular constituents and the incidence of AE-IPF and survival in a cohort of IPF patients who were diagnosed based on the official 2011 IPF ATS/ERS/JRS/ALAT Update Statement [2]. A log-rank test showed that treatment with an immunosuppressive agent after diagnosis, baseline cardiovascular diseases, higher GAP stage (≥II), higher serum LDH level (≥180 U/L), higher serum SP-D level (≥194.7 ng/mL), and higher neutrophil (≥1.77 %) and eosinophil (≥3.21 %) percentages in BAL fluid samples were associated with poor AE-free probability. In the Cox analysis adjusted for treatment with an immunosuppressive agent, baseline cardiovascular diseases, higher GAP stage (≥II), and a higher eosinophil percentage (≥3.21 %) in BAL fluid samples were predictors of an AE-IPF.

It was reported that the human lung microbiome and any changes in its numbers and composition play important roles in the pathogenesis and progression of lung diseases, including IPF [17, 18]. In addition, it was recently reported that patients with IPF had a higher bacterial load in BAL fluid compared with control subjects and that an increased bacterial load at the time of diagnosis identified patients with more rapidly progressive IPF and those at a higher risk of mortality [19]. The multicenter PANTHER trial found that combination therapy (prednisone, azathioprine, and N-acetylcysteine) was associated with more AEs than a placebo. [20]. The present study also demonstrated that treatment with an immunosuppressive agent was associated with an AE-IPF in the log-rank test and unadjusted Cox analysis. In addition, Papiris et al. recently reported that immunosuppression adversely affected the outcomes of AE-IPFs [21]. These findings imply that treatment with an immunosuppressive agent may increase the bacterial load in the lungs of patients with IPF and lead to an AE-IPF. Further studies are needed to elucidate the association between bacterial load and AE-IPFs. However, baseline cardiovascular diseases, higher GAP stage (≥II), and higher eosinophil percentage (≥3.21 %) in BAL fluid samples were predictors of an AE-IPF, even after adjustment for treatment with an immunosuppressive agent in the Cox analysis. These risk factors were thought to be independent of treatment with an immunosuppressive agent.

BAL fluid analyses in IPF patients typically show an increase in total cell count, neutrophils (>5 %), and eosinophils (>5 %) [22]. In the present study, baseline median neutrophil and eosinophil percentages in the BAL fluid of patients who experienced an AE were 7.0 % (IQR: 3.2–10.5 %) and 4.6 % (IQR: 2.1–7.0 %), respectively. These BAL fluid findings were consistent with those previously reported in patients with IPF [22]. This study showed that higher neutrophil (≥1.77 %) and eosinophil (≥3.21 %) percentages in BAL fluid samples were associated with poor AE-free probability in the log-rank test. In the adjusted Cox analysis, a higher baseline eosinophil percentage (≥3.21 %) in BAL fluid samples was a predictor of an AE-IPF. Relative increases in eosinophil and neutrophil percentages in BAL fluid may identify a subset of patients with disease that is more “active” or in an accelerated phase of tissue damage that may predispose them to an AE. Although inflammation is never a prominent histopathological finding in UIP [2], occult sustained eosinophil and neutrophil accumulations in the alveolar space may be key immune effector cells driving the inflammatory response and may play a role in the occurrence of an AE-IPF. Findings of recent studies of high BAL fluid eosinophil and neutrophil percentages in patients with IPF during AEs lend support to this possibility [3, 9]. Tabuena et al. [23] found that BAL fluid neutrophil and lymphocyte counts predicted mortality in current smokers in a study of 81 patients with IPF. Kinder et al. recently reported that each doubling of baseline BAL fluid neutrophil percentage was associated with a 30 % increase in mortality risk in a series of 156 patients with biopsy-proven IPF [24]. These observations also suggest the potential contribution of inflammation in the pathogenesis of IPF. Although the possible role of a chronic inflammatory response has been largely neglected because of the inefficacy of corticosteroids and immunosuppressants in the treatment of IPF [2, 20], further studies focusing on the potential role of inflammation in IPF pathogenesis and AE-IPF occurrence are needed. Current practice and consensus guidelines [2] do not recommend performing BAL for a determination of cellular constituents in BAL fluid at the time of IPF diagnosis. However, the indication for BAL with a cellular analysis may become clearer when effective therapies to prevent an AE-IPF are discovered.

This study showed that a baseline higher GAP stage (≥II) was one of the predictors of an AE-IPF and a significant prognostic factor for IPF in the adjusted Cox analysis. This GAP model consists of four baseline variables: gender (G), age (A), and two lung physiology variables (P) (FVC and diffusing capacity for carbon monoxide) [12]. It has been proposed that the GAP model was useful in the prediction of mortality at baseline in patients with IPF [12]. In addition, it was reported that GAP stage III IPF patients tended to have a higher incidence of AEs than stage II and stage I patients [25]. The present findings along with those obtained in previous studies suggest that the GAP staging system could be used as a quick and simple screening method for predicting AE-IPF.

Cardiovascular disease has been reported to be one of the most frequently observed comorbidities in patients with IPF and associated with shortened survival time [26, 27]. However, the impact on AE-IPF has remained unknown. This study showed that baseline cardiovascular disease was not only a prognostic factor for IPF, but also a risk factor for an AE-IPF in the adjusted Cox analysis. Further studies are needed to elucidate the mechanisms of why cardiovascular disease increases the risk of AE-IPF.

SP-D is a lipoprotein complex synthesized mainly by alveolar type II epithelial cells and is secreted into a liquid layer lining the lung epithelium. Increased serum SP-D levels obtained at the time of IPF diagnosis were reported to be associated with increased mortality [28–30]. In the present study, serum SP-D levels obtained at the time of initial diagnosis were associated with AE-free probability in a log-rank test and overall survival in the adjusted Cox analysis. Two mechanisms may contribute to elevated SP-D serum levels in pulmonary fibrosis: (1) an absolute increase in alveolar type II epithelial cells due to diffuse hyperplasia increases at the source of pulmonary SP-D; and (2) epithelial injury and basal membrane leakage may cause spillover into the circulation [28]. Evidence indicates that repetitive injuries to alveolar epithelial cells trigger an exaggerated wound healing response resulting in extensive scar formation, and finally, pulmonary fibrosis [31, 32]. Together with previous observations, the present findings suggest that injuries to alveolar epithelial cells may be one of the important triggers for IPF progression.

In a randomized trial of pirfenidone, a positive treatment effect was demonstrated, including fewer AE-IPF episodes (14 % versus none) [33]. However, a previous study did not reproduce the inhibitory effect of pirfenidone on AE-IPF [34]. In the present study, neither treatment with pirfenidone nor N-acetylcysteine was associated with AE-free probability in a log-rank test. However, this might be due to the small number of patients treated with pirfenidone or NAC in this study. Whether new antifibrotic agents have preventive effects against AE-IPF is an interesting clinical question. Further studies are needed to elucidate this question.

Previously reported conflicting results regarding risk factors for an AE-IPF were probably partly related to the use of multiple AE-IPF definitions. According to previously reported AE-IPF criteria, endotracheal aspirate or BAL fluid should be examined to exclude pulmonary infection, and patients who do not undergo these procedures should be termed as having a “suspected AE” [4]. However, these procedures are often not feasible given the significant hypoxemia typical in an AE-IPF. There is a frequent inability to exclude underlying infection results confidently in a large number of “suspected” AE-IPF cases that cannot be confirmed. Therefore, the definition of AE-IPF in the present study did not include the performance of endotracheal aspirate collection or BAL as an essential component. Collard et al. recently reported that suspected AE-IPF cases were clinically indistinguishable from definite AE-IPF cases, and were associated with similarly high risks of disease progression and short-term mortality in IPF [35]. Ryerson et al. recently proposed expanding the AE-IPF diagnostic criteria by allowing the diagnostic criteria to be met without the performance of invasive procedures (e.g. bronchoscopy) [36]. In fact, the definition of AE-IPF used in recent clinical trials of nintedanib did not include the performance of endotracheal aspirate collection or BAL as essential components, and infection was excluded in accordance with routine clinical practice and microbiologic studies [37]. Therefore, the results obtained in the present study are thought to be practical for the management of patients with IPF in daily clinical settings.

There are some limitations to this study. First, this was a retrospective study with a small number of subjects. Because of the small numbers of AEs and deaths, risk factors and prognostic factors were adjusted only by treatment with an immunosuppressive agent in the multiple Cox analysis. Because the associations among several factors seem to be complex, further study in a larger patient cohort is needed to analyze independent risk factors for AE-IPF and prognostic factors for IPF. Second, although the present study suggests that alveolar inflammation may be one of the key pathways activated prior to the development of an AE-IPF, the precise biological mechanisms of AE-IPF remain unknown. Third, this study included only Japanese patients. Therefore, further studies are needed to ascertain whether these results can be applied equally to other ethnic groups. The relative rarity of AE-IPF suggests that these research questions should be answered by multicenter collaborations.

## Conclusions

In conclusion, this study demonstrated that baseline cardiovascular diseases, higher GAP stage (≥II), and higher eosinophil percentage (≥3.21 %) in BAL fluid samples were predictors of an AE-IPF. Further studies are needed to elucidate the association between these markers and mechanisms of AE occurrence.

## Abbreviations

A-a DO_2_, alveolar-arterial difference in oxygen; AE, acute exacerbation; BAL, bronchoalveolar lavage; CI, confidence interval; CPI, composite physiologic index; CRP, C-reactive protein; DLCO, diffusing capacity for carbon monoxide; FiO_2_, fraction of inspired oxygen; FVC, forced vital capacity; GAP, gender-age-physiology; HR, hazard ratio; HRCT, high-resolution computed tomography; IPF, idiopathic pulmonary fibrosis; IQR, interquartile range; KCO, carbon monoxide transfer coefficient; KL-6, Krebs von den Lungen-6; LDH, lactate dehydrogenase; MST, median survival time; NAC, N-acetylcysteine; P/F ratio, partial pressure of oxygen in arterial blood (PaO_2_)/fraction of inspired oxygen (FiO_2_) ratio; PaO_2_, partial pressure of oxygen in arterial blood; ROC, receiver operating characteristic; SP-D, surfactant protein-D; UIP, usual interstitial pneumonia
